# Efficacy of ketamine for comorbid depression and acute or chronic pain: A systematic review

**DOI:** 10.3389/fpain.2022.1022767

**Published:** 2022-10-24

**Authors:** Aksharra Balachandran, Vanessa K. Tassone, Fathima Adamsahib, Anne-Marie Di Passa, Sarah Kuburi, Ilya Demchenko, Karim S. Ladha, Venkat Bhat

**Affiliations:** ^1^ Interventional Psychiatry Program, Mental Health and Addictions Service, St. Michael’s Hospital, Unity Health Toronto, Toronto, ON, Canada; ^2^ Department of Anesthesia, St. Michael’s Hospital, Unity Health Toronto, Toronto, ON, Canada; ^3^ Department of Anesthesiology and Pain Medicine, University of Toronto, Toronto, ON, Canada; ^4^ Institute of Medical Science, Faculty of Medicine, University of Toronto, Toronto, ON, Canada; ^5^ Department of Psychiatry, Faculty of Medicine, University of Toronto, Toronto, ON, Canada; ^6^ Li Ka Shing Knowledge Institute, St. Michael’s Hospital, Unity Health Toronto, Toronto, ON, Canada; ^7^ Krembil Research Institute, University Health Network, Toronto, ON, Canada

**Keywords:** mood disorders, pain, glutamate, dissociative anesthetics, comorbidity, ketamine

## Abstract

Pain and depression frequently co-occur. Due to its antidepressant and analgesic properties, ketamine has been used for the management of treatment-resistant depression and pain. This systematic review examined the literature on the efficacy of sub-anesthetic doses of ketamine in individuals experiencing comorbid depression and chronic pain (CDCP), as well as comorbid depression and acute pain (CDAP). A secondary objective was to provide an assessment of dosage, route, and adverse effects of ketamine treatment for CDCP and CDAP. A literature search was conducted on MEDLINE, PsycINFO, and Embase databases, coupled with a manual screening of the bibliography sections of included articles. In addition, registered ongoing and planned trials were searched on Clinicaltrials.gov. The end date of the search was April 9th, 2022. Included studies assessed changes in depression and pain in patients receiving at least one sub-anesthetic dose of ketamine. Assessment of quality was conducted using the GRADE checklist. Of the 7 CDCP clinical trials, 3 reported a reduction in depression and pain, 3 reported a reduction in depression or pain only, and 1 reported no improvement in either comorbidity. Among the 7 CDAP clinical trials, 4 studies found improvements in depression and pain while the remaining 3 reported improvements in only one parameter. Ten of the 12 case studies and 2 of the 3 observational studies assessing CDCP and CDAP found improvements in pain and depression scores post-treatment with effects of variable duration. The planned methodologies of the registered clinical trials are in line with those of the published research. Preliminary evidence supports the efficacy of ketamine in treating CDCP and CDAP. However, the current review identified a small number of heterogeneous studies with mixed results, preventing comprehensive conclusions. More longitudinal placebo-controlled studies are needed to identify the effects of ketamine for patients with CDCP and CDAP.

## 1. Introduction

Major depressive disorder (MDD) is one of the most disabling psychiatric illnesses worldwide, with a global societal cost of 65.5 million disability-adjusted life years (1). Depression accounts for approximately 50% of psychiatric consultations and 12% of all hospital admissions (2). Furthermore, 10% of the world's population suffers from chronic pain and another 10% of adult individuals are diagnosed with chronic pain annually (3). Although chronic pain, defined as persistent or recurrent pain lasting longer than three months (4, 5), is a significant health concern on its own (6), it is also a component of many chronic conditions. When co-occurring with depression (comorbid depression and chronic pain, CDCP), it represents an even greater health concern (7).

The prevalence of pain symptoms in patients with depression and of depression symptoms in patients with chronic pain is higher than the prevalence rates of both conditions alone (7, 8). Approximately 65% of patients with depression experience one or more pain complaints, and depression is present in 5% to 85% of patients with pain conditions, depending on the study setting (7). Furthermore, recognition and treatment of CDCP are more challenging than those of depression or pain alone (9). Conventional treatments for CDCP, such as monoaminergic antidepressants and psychotherapy, have a significant proportion of non-responders (10). Due to the highly disabling nature of CDCP and the associated persistent levels of daily stress, poor prognosis, and low quality of life, novel treatments for effective management of this condition are urgently needed (10).

Of similar concern is the presence of acute pain, which also commonly co-occurs with depression (comorbid depression and acute pain, CDAP). Acute pain differs from chronic pain in its duration - in particular, acute pain is present for less than 6 months (11). Previous research has found that approximately 75% of patients diagnosed with MDD presented to their general practitioner with complaints unrelated to the disorder (12). Fifty percent of these complaints were related to acute pain, including myalgia, chest, abdominal, trigeminal pain, and headaches (12). Acute pain in MDD may also be commonly seen in post-operative, post-labour, or post-caesarean contexts. Despite its prevalence, research on managing CDAP is limited (13).

Ketamine has been used as an anesthetic agent for over 50 years. More recently, sub-anesthetic doses of ketamine have been shown to exert antidepressant and analgesic properties in the management of treatment-resistant depression (TRD) (14, 15) and chronic pain (16, 17), respectively. There is a substantial overlap in the neurobiology of depression and pain, with both being characterized by the disruption of sensory, emotional, and cognitive neuronal circuits (18–20). Additional evidence suggests that depression and pain have overlapping descending pathways in the central nervous system, such as the pain suppression pathway mediated through the projections to the periaqueductal gray matter of the upper brainstem (21). While antidepressants provide pain relief by modulating these descending pathways and increasing serotonin and norepinephrine levels in the synapse (22), sub-anesthetic doses of ketamine, a non-competitive N-methyl-D-aspartate (NMDA) receptor antagonist, increases the glutamatergic activity of the brain. This could impact neural signaling, plasticity, and connectivity, leading to enhanced synaptogenesis and decreased levels of pain and depression (17, 23).

A number of systematic reviews and meta-analyses have examined the role of sub-anesthetic doses of ketamine in the treatment of depression (24–29) and have demonstrated that it is a promising novel agent for the management of unipolar depressive symptoms (23). Research has also shown that antidepressant effects of ketamine are observed within hours, making it an advantageous treatment option due to its rapidity. This is true particularly in cases of TRD and suicidality, where the therapeutic lag time associated with traditional antidepressants may not be acceptable (23).

Although ketamine has not been formally approved by the North American federal agencies (e.g., U.S. Food and Drug Administration, Health Canada) as a treatment modality for pain, it has been used to treat post-operative pain, chronic pain, complex regional pain syndrome, phantom limb pain, and other neuropathic conditions requiring analgesia (30). In systematic reviews and meta-analyses on the role of ketamine in the treatment of chronic pain, ketamine has shown beneficial effects lasting from 12 weeks to 6 months (17, 31). Furthermore, previous systematic reviews and meta-analyses have found ketamine to be a promising alternative treatment for acute pain, particularly in emergency settings (32, 33).

Despite previous efforts to systematically review the role of ketamine in the treatment of depression or pain (i.e., acute and chronic) individually (17, 24–29, 31, 33), there remains uncertainty regarding how this compound affects patients with CDCP and CDAP. To our knowledge, this systematic review is the first to examine the efficacy of ketamine for the treatment of patients who were experiencing depression and chronic or acute pain concurrently, CDCP and CDAP. Although IsHak et al. (34) conducted a systematic review on the general treatment approaches for CDCP, the paper did not systematically review the effects of ketamine. Schoevers et al. (35) contributed to this growing field by providing a review on the effectiveness of a less common oral administration of ketamine for CDCP treatment. The final search date of this review was in 2014, and thus, an updated review is needed to explore the latest advancements. In contrast, there are no systematic reviews on the management of CDAP. Complementary to the published literature, reviewing registered (i.e., ongoing and planned) clinical trials involving the use of ketamine for acute and chronic pain would provide a more comprehensive understanding of the clinical undertakings in the field.

## 2. Methods

This systematic review followed the Preferred Reporting Items for Systematic Reviews and Meta-Analyses (PRISMA) guidelines (36). The completed PRISMA checklist can be found in the Supplementary Materials.

### 2.1. Search strategy

#### 2.1.1. Published studies

Relevant studies published before April 9, 2022 were identified using the MEDLINE, PsycINFO, and Embase OVID databases. The search strategy was based on a combination of Medical Subject Headings terms including “ketamine”, “S-ketamine”, as well as indexed terms related to depression and pain: (depression OR mood disorders OR major depressive disorder OR depress* OR affective disorders) AND (pain OR neuralgia OR postoperative OR cesar* section OR caesar* section OR pain*) AND [ketamine(mh) OR S-Ketamine OR Esketamine]. The complete search strategy is provided in the Supplementary Materials. Potentially relevant papers were first identified through title and abstract searches. The full text of the articles that were eligible for inclusion were subsequently reviewed. A manual search through the references section of included studies was additionally performed. Two independently working authors, A.B. and F.A. or V.K.T., carried out the search and screening process. Discrepancies were discussed, consulted with V.B., and resolved by consensus.

#### 2.1.2. Registered ongoing and planned clinical trials

A search of past and ongoing clinical trials was conducted using Clinicaltrials.gov (https://clinicaltrials.gov/) using the terms: *ketamine AND (depression OR postpartum depression) AND (pain OR cancer OR surgery)*, with no restrictions on the status of the study in the search. The complete search strategy is provided in the Supplementary Materials. The end date of the search was April 9, 2022.

### 2.2. Exclusion and inclusion criteria

Articles that were excluded were animal studies, review articles, and papers written in a language other than English. In addition, duplicate results were removed. There were no restrictions on participant characteristics, such as sex/gender or age. Inclusion criteria for this review were either studies that had participants diagnosed with MDD by a psychiatrist according to the *Diagnostic and Statistical Manual of Mental Disorders—Fourth or Fifth Edition* or studies in which participants were evaluated for depression using a valid rating scale and required to meet the minimum cut-off score. In addition, the inclusion criteria required participants of selected studies to have either chronic or acute pain conditions which were being treated with ketamine and were measured by pain rating scales at baseline and as treatment with ketamine progressed. Chronic pain was classified as the presence of pain for greater than 6 months, while acute pain was described as pain present for less than 6 months. Studies included in this review must have reported and/or analyzed changes in depression and pain in patients who met the above criteria for each condition simultaneously and received at least one sub-anesthetic dose of ketamine, regardless of administration route. Published studies included in the review were categorized into clinical trials, observational studies, and case reports/series. To ensure the included registered (i.e., ongoing and planned) clinical trials captured participants with depression, studies were excluded if the inclusion criteria did not require participants to have a depression diagnosis or symptoms as indicated by a valid rating scale.

### 2.3. Data extraction

The following information was extracted from each published study by two independent reviewers (A.B. and F.A.) using a standardized format: first author, year of publication, clinical diagnosis, participant characteristics (mean age, percentage female), as well as sample size and study design. In regard to the administration of treatment, the following data were extracted: administration route (intravenous or oral), dose, and treatment duration. The data on outcome parameters related to measurement of depression and pain, treatment response, and remission rate, as well as adverse effects and study limitations were also extracted. In addition, data regarding sample size, clinical diagnosis, study design, treatment route and dose, as well as start, registration, and completion dates were extracted from the registered (i.e., ongoing and planned) clinical trials.

### 2.4. Quality assessment

The quality of the studies was assessed according to the Grading of Recommendations, Assessment, Development and Evaluations (GRADE) checklist (37, 38), which evaluates the criteria of selection, performance, detection, and reporting biases. The GRADE also verifies the objectivity and selectivity of the reported outcomes and the consistency between planned and actual study endpoints. Supplementary Table 1 summarizes the results of the quality assessment.

Selected placebo-controlled clinical trials were assessed to have a low risk for selection and performance biases, since 93% of them (39–50) used a randomized double-blind treatment protocol. All studies reported adequate sequence generation. The included open-label study (51) is at a higher risk of performance bias due to the lack of blinding, while selection bias and detection bias cannot be adequately reported. However, all participants received the same treatment, which was aimed at attempting to minimize the potential selection bias. Selection, detection, and performance bias are not applicable to case studies or observational studies which included retrospective designs. In all but one study (39), a minimum of 80% of participants enrolled in the studies completed the trial as per the protocol, thus reducing reporting bias. We identified reports of both significant and nonsignificant findings, demonstrating a low selective reporting bias.

## 3. Results

### 3.1. Study selection

**Figure 1:**
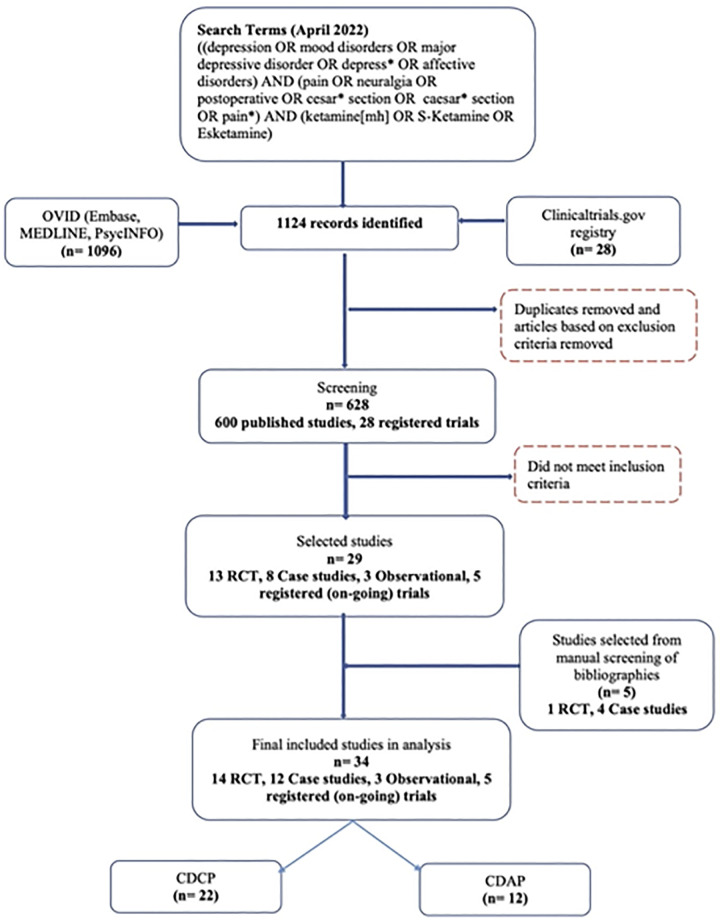
PRISMA flow chart reflecting the search strategy and screening process of published studies and registered (i.e., ongoing and planned) clinical trials included in the systematic review. CDAP, comorbid depression and acute pain; CDCP, comorbid depression and chronic pain; RCT, randomized-controlled trial.

The initial search yielded 1096 published papers and 28 registered (i.e., ongoing and planned) trials. Of these, 496 published articles were removed based on the exclusion criteria. This resulted in a total of 628 studies (600 published articles and 28 registered trials) that were examined for their titles and abstracts, as well as the full text, depending on their potential eligibility. Five additional studies were qualified based on the manual search of the references within eligible studies. At the full-text level, 599 studies (576 published articles and 23 registered trials) did not meet the inclusion criteria. This resulted in the final number of 29 published studies included in the present review (14 clinical trials (39–52), 3 observational studies (53–55), and 12 case studies (56–67)), as well as 5 registered (i.e., ongoing and planned) clinical trials (68–72). Figure 1 illustrates the PRISMA (36) flow chart for this systematic review.

### 3.2. Study types and distribution

**Figure 2:**
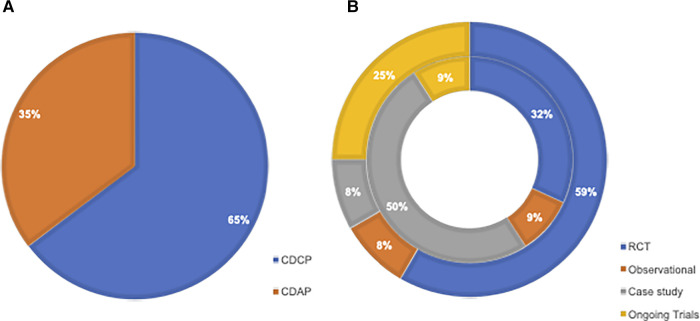
(**A**) Pie chart illustrating the breakdown of studies looking at CDCP vs. CDAP. (**B**) Sunburst chart illustrating the distribution of selected studies, including RCTs, observational, case studies, and registered (i.e., ongoing and planned) trials separated into those looking at CDAP (outer segment) and CDCP (inner segment). CDAP, comorbid depression and acute pain; CDCP, comorbid depression and chronic pain; RCT, randomized-controlled trial.

Across published studies and registered trials, a total of 34 studies were identified. Twenty-two (65%) assessed CDCP and 12 (35%) assessed CDAP (Figure 2A).

#### 3.2.1. CDCP

When looking at the distribution of study types among those concerning CDCP, 7 out of 22 (32%) were published clinical trials, 2 out of 22 (9%) were observational studies, 11 out of 22 (50%) were case studies, and 2 out of 22 (9%) were registered clinical trials.

#### 3.2.2. CDAP

When looking at the distribution of study types among those concerning CDAP, 7 out of 12 (59%) were published clinical trials, 1 out of 12 (8%) was an observational study, 1 out of 12 (8%) was a case study, and 3 out of 12 (25%) were registered clinical trials. The distribution of study types between CDCP and CDAP is illustrated in Figure 2B.

### 3.3. Characteristics of included studies

The 29 published studies included 3,500 patients assigned to various ketamine regimens including racemic ketamine, esketamine, or treatment as usual. Overall, the sample sizes of the clinical trials and observational studies ranged from *N* = 16 to *N* = 654 (mean *N* = 205, *SD* = 167): specifically, ten studies had sample sizes over 100 (39, 41–43, 45, 47, 49, 50, 54, 55), three studies had sample sizes between 50 and 100 (46, 48, 52), and the remaining four studies had sample sizes of less than 50 people (40, 44, 51, 53). The clinical trials and observational studies that examined CDCP had varying sample sizes, with the smallest having *N* = 16 and the largest having *N* = 417 participants. The clinical trials and observational studies examining CDAP had samples between *N* = 90 and *N* = 654 participants. One case series (61) described treatment of two patients, whereas the remaining case studies outlined individual cases. Characteristics such as mean age, percentage female, dose and route of administration, participant diagnosis, and study outcomes and measures are presented in Tables 1A,B, 2.

**Table 1A:** Selected characteristics of clinical trials and observational studies pertaining to CDCP.

	Corriger et al. (54)	Falk et al. (53)	Fallon et al. (39)
Year (*N*)	2022 (*256*)	2020 (*16*)	2018 (*214*)
Mean Age (Female%)	51 (76%)	53 (57%)	58 (66%)
Study duration	1 year	5 days	2 weeks
Study design	Observational study	Placebo controlled retrospective study	Double-blind RCT of ketamine vs placebo
Dose (route)	Cumulative dose between ≤100 mg and ≥270 mg over 1–≥6 days (intravenous or oral)	0.25 mg/kg (intravenously, esketamine)	40 mg/d–400 mg/d (oral)
Diagnosis	Patients attending pain clinics for refractory chronic pain	Patients in palliative care with different types of cancer	Cancer-related neuropathic pain
Outcome measures	NRS (pain) HADS (anxiety and depression)	STADI (depression) NRS (pain)	MPQ (pain) HADS (anxiety and depression)
Findings	1. NRS score decreased at one week after ketamine, 6 months, and 12 months (*p *<0.001). 2. Anxiety score decreased over time at one week, 6 months and 12 months (*p *< 0.001). 3. Depression subscale of HADS decreased at one week, 6 months, and 12 months (*p *< 0.001).	1. No significant reductions in pain (*p *= 0.75) or depression symptoms (*p *= 0.23) were found when the ketamine group was compared to the placebo group.	1. Ketamine was equivalent to placebo for cancer-related neuropathic pain. 2. No differences between the groups on HADS scores.
Adverse effects	• Not reported	• Not reported	• Cognitive disturbance • Dizziness • Fatigue • Nausea • Somnolence
Limitations	• Observational design has known biases, lack of a control or placebo and missing data linked to unanswered phone calls during follow-ups • No standardized ketamine dose	• Small sample size • Retrospective data prevented randomization • Low single treatment dose	• Short study duration • Low treatment dosage • Low compliance (23%) • Lack of statistical data

	Jafarinia et al. (40)	Liu et al. (42)	Mitchell and Fallon (44)
Year (*N*)	2016 (*40*)	2021 (*303*)	2002 (*38*)
Mean Age (Female%)	40 (75%)	47 (100%)	71 (43%)
Study duration	6 weeks	3 months	4–11 days
Study design	Double-blind RCT of ketamine vs diclofenac	Double-blind RCT of ketamine vs placebo with 3 groups: racemic ketamine, esketamine and control group	Double-blind RCT of ketamine vs placebo
Dose (route)	150 mg/d (oral)	0.125 mg/kg (intravenously)	0.6 mg/kg (intravenously)
Diagnosis	CDCP	Breast cancer patients with MDD	Patients with critical limb ischemia
Outcome measures	HDRS_17_ and HADS (depression) VAS (pain)	HDRS_17_ (depression) VAS (pain) Serum BDNF and 5-HT	BPI (pain) HADS (depression)
Findings	1. No differences between the groups on HDRS_17_ scores at 3 weeks post-intervention (*p *= 0.12). 2. Ketamine group had lower HDRS_17_ scores at 6 weeks post-intervention (*p *= 0.02). 3. HADS scores were lower in the ketamine group at 3 and 6 weeks (*p *= 0.001 and *p *= 0.01, respectively). 4. No differences between the groups on VAS scores at 3 and 6 weeks (*p *= 0.69 and *p *= 0.70).	1. At 1 and 3 days post-surgery, VAS scores were lower (*p *< 0.001) in racemic and esketamine groups, compared to the control group. 2. No difference in VAS scores between the groups at 1 week after surgery (*p *= 0.413). 3. At 3 days, 1 week, and 1 month post-surgery HDRS_17_ scores were lower (*p *< 0.001) in racemic and esketamine groups, compared to the control group. 4. No difference in HDRS_17_ scores between the groups at 3 months (*p *= 0.05). 5. At 3 days, 1 week, and 1 month serum levels of BDNF were higher (*p *< 0.001) in racemic ketamine and esketamine groups, compared to the control group. 6. At 3 days, 1 week, and 1 month serum levels of 5-HT were higher (*p *< 0.001) in racemic ketamine and esketamine groups, compared to the control group.	1. Within the first 24 h post-infusion, ketamine group reported greater pain relief (*p *< 0.05). 2. Within the first 24 h, the ketamine group reported greater improvements of pain on their general activity (*p *= 0.03) and on enjoyment of life (*p *= 0.004). 3. No significant differences between the group on HADS scores.
Adverse effects	• Blurred vision • Restlessness • Nervousness • Tremor • Loss of appetite	• Not reported	• Ketamine group reported feeling more emotional
Limitations	• Lack of placebo group • Small sample size • No reports on means and *SD* values	• Low treatment dose	• Small sample size • Short study duration • No reports of *SD* scores • No reports of mean HADS scores and *p*-values
	Sorel et al. (51)	Wang et al. (45)	Xu et al. (46)
Year (*N*)	2018 (*19*)	2020 (*417*)	2017 (*50*)
Mean Age (Female%)	63 (79%)	48 (100%)	43 (100%)
Study duration	5 days	7 days	8 days
Study design	Open-label CT with ketamine infusions	Double-blind RCT with four groups: racemic ketamine, low- and high-dose of esketamine, and control group	Double-blind RCT of ketamine vs placebo
Dose (route)	0.7–1.0 mg/kg/day (intravenously, for 5 days)	0.25/0.5 mg/kg (intravenously)	0.5 mg/kg (intravenously)
Diagnosis	Complex regional pain syndrome	Cervical carcinoma patients with MDD	Breast cancer patients with MDD
Outcome measures	VAS and NPSI (pain) HADS, MADRS, and BDI (depression)	HDRS_17_ (depression) VAS (pain) Serum BDNF and 5-HT	HDRS_17_ (depression) VAS (pain)
Findings	1. Measures of VAS scores during rest and activities were reduced (*p *< 0.0001) in comparison with the scores before treatment. 2. Total score on NPSI was improved (*p *< 0.0001). 3. Reductions in all depression measures: HADS (*p *< 0.0001), MADRS (*p *< 0.0001), and BDI (*p *= 0.0007).	1. At 1, 2, and 3 days post-surgery, VAS scores were lower (*p *< 0.05) in all treatment groups when compared to placebo, with the high esketamine group showing larger reductions than other treatment groups (*p *< 0.05). 2. At 5 days post-surgery, VAS scores were returned to the baseline in all groups. 3. At 1, 2, and 3 days post-surgery, HDRS_17_ scores were lower (*p *< 0.05) in all treatment groups when compared to placebo, with the high esketamine group showing the greatest reductions (*p *< 0.05). 4. No differences in HDRS_17_ were found between the groups at 5 days post-surgery. 5. At 1, 2, and 3 days all treatment groups showed higher (*p *< 0.001) BDNF levels than the controls: racemic ketamine, low dose esketamine, and high dose esketamine. 6. At 1, 2, and 3 days, all treatment groups showed higher (*p *< 0.001) 5-HT levels than the controls: racemic ketamine, low dose esketamine, and high dose esketamine. 7. No differences among the 4 groups on BDNF and 5-HT levels at day 7.	1. At 1, 3, and 7 days post-surgery, HDRS_17_ were lower (*p *< 0.05) in the ketamine group, in comparison to before surgery. 2. At 1 and 3 days post-surgery HDRS_17_ were lower (*p *< 0.05) in the ketamine group, in comparison to the control group; no difference between the groups at 7 days post-surgery. 3. At 1, 3, and 7 days post-surgery, no differences among the groups were found on measures of VAS.
Adverse effects	• Moderate asthenia • Variations of heart rate and BP • Headache • Drowsiness/fatigue	• Nausea • Dizziness • Vomiting	• Nausea • Dizziness • Headache • Euphoric mood
Limitations	• Small sample size • Short study duration • Lack of placebo group	• Short study duration • No reports of mean and *SD* scores for pain and depression measures	• Short study duration • Small sample size

5-HT, 5-hydroxytryptamine; BDNF, Brain-derived Neurotrophic Factor; BDI, Beck Depression Inventory; BP, Blood pressure; BPI, Brief Pain Inventory; CDCP, Comorbid Depression Chronic Pain; CT, Clinical Trial; HADS, Hospital Anxiety and Depression Scale; HDRS_17_= Hamilton Depression Rating Scale (17 item-long); MADRS, Montgomery-Åsberg Depression Rating Scale; MDD, Major Depressive Disorder; MPQ, McGill Pain Questionnaire; NPSI, Neuropathic Pain Symptom Inventory; NRS, Numerical Rating Scale; RCT, Randomized Controlled Trial; *SD*, Standard Deviation; STADI, State-Trait Anxiety Depression Inventory; VAS, Visual Analog Scale.

**Table 1B:** Selected characteristics of clinical trials and observational studies pertaining to CDAP.

	Han et al. (50)	Jiang et al. (41)	Kudoh et al. (52)
Year (*N*)	2022 (*275*)	2016 (*120*)	2002 (*95*)
Mean Age (Female%)	32 (100%)	42 (44%)	47
Study duration	28 days	5 days	3 days
Study design	Double-blind RCT of ketamine vs in patient-controlled intravenous analgesia control	Double-blind RCT of ketamine vs placebo	RCT of ketamine vs placebo with 3 groups: MDD patients + ketamine (group A), MDD patients + placebo (group B), and control group + ketamine (group C)
Dose (route)	0.5 mg/kg (intravenously, esketamine)	0.75 mg/kg (intravenously)	1.0 mg/kg (intravenously)
Diagnosis	Pregnant women undergoing caesarean section	Orthopedic surgery patients	MDD patients undergoing orthopedic surgery
Outcome measures	EPDS (postpartum depression) VAS (pain)	PHQ_9_ (depression) VAS (pain) Serum BDNF	HDRS_21_ (depression) VAS (pain)
Findings	1. Rate of depression significantly lower in esketamine group at 3 and 14 days after cesarean section (*p *< 0.05). 2. EPDS significantly lower in esketamine group at 3 and 14 days postpartum (*p*'s < 0.001); EPDS not lower at day 28. 3. VAS significantly lower in the esketamine group at 4, 8, 12, and 24 h after cesarean section (*p*'s < 0.05).	1. VAS scores were lower in the ketamine group at 1 and 5 days post-surgery (*p *< 0.01 and *p *< 0.01, respectively). 2. PHQ_9_ scores were lower in the ketamine group at 1 and 5 days post-surgery (*p *= 0.04 and *p *= 0.03, respectively). 3. Serum BDNF levels were higher in the ketamine group on both days (*p *= 0.02 and *p *= 0.03 at day 1 and 5, respectively).	1. HDRS_21_ score in group A was lower than in group B at day 1 post-surgery (*p *< 0.05). 2. No differences between the group A and B in HDRS_21_ scores at 3 days post-surgery. 3. VAS scores in group A at 8 and 16 h post-surgery were lower than Group B (*p *< 0.05). 4. No differences in VAS scores between group A and B at day 1 post-surgery.
Adverse effects	• Headache • Nausea • Dizziness • Drowsiness • Vomiting	• Nausea • Insomnia	• Short post-operative confusion
Limitations	• Small sample only including Chinese adults from a single center • Dose of 0.5 mg/kg lower than the subanesthetic dose for treatment of depression • Cannot determine long-term effects	• Short study duration • No diagnosis of mental disorder	• Short study duration • Lack of baseline pain assessment • No reports of the exact *p*-values • No reports of *SD* for pain scores • Missing reports of mean values for depression scores

	Ma et al. (43)	Wang et al. (48)	Wang et al. (55)
Year (*N*)	2019 (*654*)	2019 (*90*)	2022 (*240*)
Mean Age (Female%)	Mean age not reported (100%)	40 (69%)	30 (100%)
Study duration	2 months	7 days	3 months
Study design	Double-blind RCT of ketamine vs placebo	Double-blind RCT of ketamine vs placebo	Retrospective cohort study with control, low-, and high-dose groups
Dose (route)	0.5 mg/kg (intravenously during operation plus 160 mg post-operation *via* patient-controlled intravenous analgesia)	0.4 mg/kg (intravenously)	0.2–0.5 mg/kg (intravenously, esketamine)
Diagnosis	Pregnant women undergoing caesarean section	Post-operative bariatric surgery patients	Pregnant women undergoing caesarean section
Outcome measures	EPDS (postpartum depression) NRS (pain)	VAS and MPQ (pain) BDI and MADRS (depression)	NRS (pain) EPDS (postpartum depression) GAD_7_ (postpartum anxiety)
Findings	1. EPDS score at postpartum day 4 was lower in the ketamine group (*p *= 0.01). 2. At day 4, prevalence of postpartum blues was lower in the ketamine group (*p *= 0.02). 3. There were no differences between the groups on EPDS score at postpartum day 42 (*p *= 0.07). 4. At day 42, prevalence of PPD was lower in the ketamine group (*p *= 0.02). 5. NRS scores at day 1 and 2 post-surgery were not different between the groups (*p *= 0.20 and *p *= 0.67, respectively).	1. VAS score decreased over time (time effect *p *< 0.001) but no significant difference between groups were seen (group-by-time interaction *p *= 0.966). 2. MPQ demonstrated a significant decrease in ketamine group on post-operation day 2. 3. Change in BDI decreased over time (time effect *p *< 0.001) but no significant differences between groups (*p *= 0.389). 4. MADRS values decreased over time but no significant differences between groups (*p *= 0.674).	1. NRS (at rest) at 2, 4, 8, 24, and 48 h postpartum lower in the esketamine group (*p *= 0.021, *p *= 0.002, *p *< 0.001). 2. NRS (while coughing) was similar between ketamine and control groups within 2 h (*p *= 0.108); lower in the esketamine group at other time points (*p *= 0.031, *p *= 0.005, *p *= 0.017, *p *= 0.007). 3. EPDS not significantly different at antenatal day 1 (*p *= 0.544); EPDS lower in the esketamine group within 3 months postpartum (*p *< 0.001). 4. Morphine consumption within 24 h postpartum lower in the esketamine group (*p *< 0.001). 5. GAD_7_ score lower in the esketamine group within 1 week (*p *< 0.001). esketamine group (*p *= 0.001). 6. NRS (at rest) at 24 and 48 h postpartum lower in high-dose group than low-dose group (*p *< 0.001 and *p *= 0.011, respectively); NRS (while coughing) at 24 h postpartum lower in the high-dose group than low-dose group; no significant difference between 2 subgroups in postpartum EPDS.
Adverse effects	• Vomiting • Dizziness • Hallucinations • Nystagmus	• Nausea • Dizziness • Dysphoria • Visual hallucination	• Nausea • Vomiting • Dizziness • Pruritus
Limitations	• Study with healthy participants • Short-term pain assessment (up to 2 days post-surgery) • No reports of *SD* for pain scores	• Baseline MADRS and BDI were low indicating a lack of severe depression symptoms	• Time difference between the control and esketamine group • Doses in the subgroup dose analysis mainly dependent on body weight perhaps leading to selection bias • Retrospective study • No data on spousal relationship, family income, and domestic violence
	Xu et al. (47)	Yao et al. (49)
Year (*N*)	2017 (*330*)		2020 (*330*)
Mean Age (Female%)	32 (100%)		30 (100%)
Study duration	6 weeks		4 weeks
Study design	Double-blind RCT of ketamine vs placebo		Double-blind RCT of ketamine vs placebo
Dose (route)	0.25 mg/kg (intravenously)		0.25 mg/kg (intravenously)
Diagnosis	Pregnant women undergoing caesarean section		Pregnant women undergoing caesarean section
Outcome measures	EPDS (postpartum depression) NRS (pain)		EPDS (postpartum depression) NRS (pain)
Findings	1. No differences on EPDS scores or the prevalence of PPD between the groups at any of the time points (*p *= 0.97 and *p *= 0.90). 2. At 3 days postpartum, there were no differences between the groups on NRS scores. 3. At 6 weeks postpartum, NRS scores were lower in the ketamine group (*p *= 0.01).		1. Significant difference in degree of postpartum depression symptoms between ketamine and placebo group at 1 week postpartum (*p *= 0.029). 2. No difference was found between subjects in two groups at 2 weeks and 1 month postpartum (*p *= 0.209 and *p *= 0.319). 3. NRS score of wound pain and uterine contraction pain was lower in the ketamine group at 2 days postpartum (*p *< 0.001).
Adverse effects	• Hallucinations • Dizziness • Headache • Vomiting • Drowsiness • Diplopia		• Hallucinations • Dizziness • Headache
Limitations	• Low treatment dose • No reports of *SD* scores • No baseline assessment • Study with healthy participants		• Low treatment dose • No baseline assessment • Study with healthy participants

BDNF, Brain-derived Neurotrophic Factor; BDI, Beck Depression Inventory; EPDS, Edinburgh Postnatal Depression Scale; GAD_7_, Generalized Anxiety Disorder (7 item-long); HDRS_21_, Hamilton Depression Rating Scale (21 item-long); MADRS, Montgomery-Åsberg Depression Rating Scale; MDD, Major Depressive Disorder; MPQ, McGill Pain Questionnaire; NRS, Numerical Rating Scale; PHQ_9_, Patient Health Questionnaire (9 item-long); PPD, Postpartum Depression; RCT, Randomized Controlled Trial; *SD*, Standard Deviation; VAS, Visual Analog Scale.

**Table 2:** Selected characteristics of case studies.

Author (year)	Age (sex)	Diagnosis	Treatment protocol	Findings	Outcome measures	Adverse effects
CDCP						
Barbosa et al. (56)	65 (male)	Patient with cancer and MDD	0.5 mg/kg subcutaneous esketamine, twice weekly	1. Pain response was observed, but without stability. 2. Patient experienced a progressive relief of mood symptoms and achieved remission after the third dose.	VAS (pain) MADRS (depression)	Moderate dissociative symptoms and somnolence
Bigman et al. (57)	47 (male)	CDCP	0.5 mg/kg ketamine intramuscularly (single infusion)	1. Improvements in depression symptoms and pain levels lasting for 30 days post-infusion.	PHQ_9_ (depression) Subjective pain scores	Not reported
Hanna et al. (58)	31 (female)	Chronic pain	10 days treatment with 200 mg/day of IV ketamine, increased progressively to 800 mg/day	1. Little improvements in the first 10 days. During the 1 month follow up, the pain scores and depression symptoms were drastically improved.	VAS (pain) GAD_7_ CESD-R (depression)	No side effects were observed
Mandyam and Ahuja (59)	30 (female)	CDCP	IV ketamine, infused cumulatively over the 3 days, 0.18 mg/kg/h increased progressively to 0.45 mg/kg/h	1. Within the first 2 days, improvements in pain reduction have been observed. 2. At 3 days after the start of treatment, manic symptoms were observed. Treatment was discontinued.	Subjective pain scores Depression assessed *via* psychiatric assessment	• Visual hallucinations • Pressured speech • Delusions • Persistent psychotic thoughts • Euphoric mood
McNulty and Hahn (60)	44 (male)	CDCP and anxiety	Single subcutaneous injection with 0.5 mg/kg ketamine, subsequently 40 mg/day of oral ketamine	1. Ketamine injection provided dramatic relief in depression symptoms and pain. These good outcomes could be sustained with the oral compound.	Depression assessed *via* psychiatric assessment Subjective pain scores	Treatment was well-tolerated
Mischel et al. (61)	81 (male)	CDCP with anxiety and psychotic features	0.3–0.4 mg/kg/h of IV ketamine for 72 h	1. At 2 days after onset of the treatment, patient reported lack of suicide ideations and improvements in affect that lasted until 3 months follow-up.	Depression assessed *via* psychiatric assessment Pain measures not mentioned	Not reported
	77 (male)	Severe depression and suicide attempt, resulting in bilateral orbit trauma	0.3–0.4 mg/kg/h of IV ketamine for 1 week	1. The patient reported reduction in pain levels. Affect and personal engagement improved significantly.	Depression assessed *via* psychiatric assessment Subjective pain scores	Not reported
Rodríguez-Mayoral et al. (63)	39 (female)	Patient with cancer CDCP and suicide attempts	Single injection of 0.5 mg/kg IV ketamine	1. Depression symptoms decreased by 17% on day 1, 39% on day 3 and 72% on day 17. 2. Suicidal ideation disappeared. 3. Pain was reduced.	BEDS (depression) ESAS (pain)	Patient reported no side effects
Sexton et al. (64)	64 (male)	Patient with cancer and MDD	0.2 mg/kg/h of IV ketamine, for 26 h continuously	1. At 26 h after initiation of infusion, the patient achieved a pain score of 0/10 and mood was improved.	PHQ_9_ (depression) Subjective pain scores	Not reported
Stefanczyk-Sapieha et al. (65)	50 (male)	Patient with cancer and MDD	0.5 mg/kg IV ketamine infusion, repeated after 10 days	1. Initial beneficial effects on depression symptoms and pain that started to wear off after 72 and 24 h post first and second infusion, respectively.	BDI and HDRS (depression) ESAS (pain)	Single brief episode of a visual hallucination
Weber et al. (66)	14 (female)	Patient with depression, anxiety, and CRPS	0.12–0.56 mg/kg/h of IV ketamine for 6 days	1. Remarkable improvements in pain scores and depression symptoms. The beneficial effects could be sustained for 5 months.	NRS (pain) Depression assessed *via* psychiatric assessment	Not reported
Zanicotti et al. (67)	36 (female)	Patient with cancer and MDD	1 mg/kg of intramuscular ketamine repeated, approximately weekly	1. Rapid onset of pain relief, with variable durability. 2. Improvement in depression symptoms that started to wear off after 6–7 days.	CPS (pain) MADRS and HADS (depression)	Dissociative symptoms
CDAP						
Nichols et al. (62)	27 (male)	Patient with severe buttock pain and opioid-induced depressive disorder	19 mg/kg IV ketamine, infused cumulatively over 4 days	1. Initial improvements in pain and mood. At day 4, patient started to show manic symptoms and treatment was discontinued.	Subjective pain scores Depression assessed *via* psychiatric assessment	• Inappropriate sounds • Sexual hyperactivity • Delusional beliefs • Disinhibition • Diminished need for sleep

BDI, Beck Depression Inventory; BEDS, Brief Edinburgh Depression Scale; CDCP, Comorbid Depression Chronic Pain; CESD-R, Center for Epidemiologic Studies Depression-Revised; CPS, Comparative Pain Scale; CRPS, Complex Regional Pain Syndrome; ESAS, Edmonton Symptom Assessment System; GAD_7_, Generalized Anxiety Disorder (7 item-long); HADS, Hospital Anxiety and Depression Scale; HDRS, Hamilton Depression Rating Scale; IV, Intravenous; MADRS, Montgomery-Åsberg Depression Rating Scale; MDD, Major Depressive Disorder; NRS, Numerical Rating Scale; PHQ_9_, Patient Health Questionnaire (9 item-long); VAS, Visual Analog Scale.

### 3.4. Administration route and dosage

Overall, 23 out of 29 (79%) published studies reported administering intravenous ketamine infusions.

#### 3.4.1. CDCP

Of the seven clinical trials conducted among patients suffering from chronic pain symptoms and depression, three (43%) (42, 45, 46) administered ketamine intravenously for a surgical procedure after analgesia induction (dose range = 0.125–0.5 mg/kg). Two (29%) studies administered higher single (0.6 mg/kg dose) (44) or repeated intravenous infusions (0.7–1.0 mg/kg/day dose) (51). An additional two out of seven (29%) clinical trials reported treatment with repeated use of oral ketamine: 150 mg/day for six weeks (40) and 40–400 mg/day for two weeks (39). One observational study described results of a single dose (0.25 mg/kg) of intravenous esketamine infusion (53), while another examined a variety of patients who received cumulative doses between ≤100 mg and ≥270 mg over 1–≥6 days (intravenous or oral) (54). Among the case studies, 8 out of 11 (73%) reported treatments with repeated or continuous ketamine infusions or injections ranging from 0.12 mg/kg/h to 800 mg/day. Two case studies (18%) applied treatment with a single infusion of 0.5 mg/kg of ketamine administered intramuscularly (57) or intravenously (63). Lastly, one case study (9%) (60) reported a combination treatment of a single subcutaneous ketamine injection (0.5 mg/kg) and oral ketamine (40 mg/day, applied until the death of the patient).

#### 3.4.2. CDAP

All clinical trials with patients undergoing surgery and experiencing acute pain applied a single injection of ketamine ranging from 0.20 mg/kg to 1.0 mg/kg, one of which also administered 160 mg post-operation *via* patient-controlled intravenous analgesia (43). One observational study (55) applied esketamine doses ranging from 0.2 mg/kg to 0.5 mg/kg intravenously. Only one case study (62) administered a ketamine infusion of 19 mg/kg over 4 days in an acute pain setting. Treatment was discontinued on day 4 when the patient developed manic symptoms.

### 3.5. Outcome measures

The published studies were characterized by high heterogeneity in terms of outcome measures. The most common scales used to assess depression symptoms across published studies were the Hamilton Depression Rating Scale (HDRS) (*N* = 6) (40, 42, 45, 46, 52, 65) and Hospital Anxiety and Depression Scales (HADS) (*N* = 6) (39, 40, 44, 51, 54, 67). The Edinburgh Postnatal Depression Scale (EPDS) (*N* = 5) (43, 47, 49, 50, 55) and Montgomery-Åsberg Depression Rating Scale (MADRS) were also frequently used (*N* = 4) (48, 51, 56, 67). The Visual Analogue Scale (VAS) was the most frequently administered across published studies for pain measurement (*N* = 11) (40–42, 45, 46, 48, 50–52, 56, 58), followed by the Numerical Rating Scale (NRS) (*N* = 7) (43, 47, 49, 53–55, 66). Of note, 50% of all case studies (57, 59–62, 64) reported the use of subjective measures to quantify pain symptom severity following administration of treatment.

#### 3.5.1. CDCP

Studies assessing CDCP used the following scales to measure depression scores: HADS, HDRS, Beck Depression Inventory (BDI), MADRS, State-Trait Anxiety Depression Inventory (STADI), and Center for Epidemiological Studies Depression-Revised (CESD-R). The following scales were used to measure pain scores: VAS, NRS, Brief Pain Inventory (BPI), Comparative Pain Scale (CPS), Neuropathic Pain Symptom Inventory (NPSI), McGill Pain Questionnaire (MPQ) and Edmonton Symptom Assessment System (ESAS).

#### 3.5.2. CDAP

**Figure 3:**
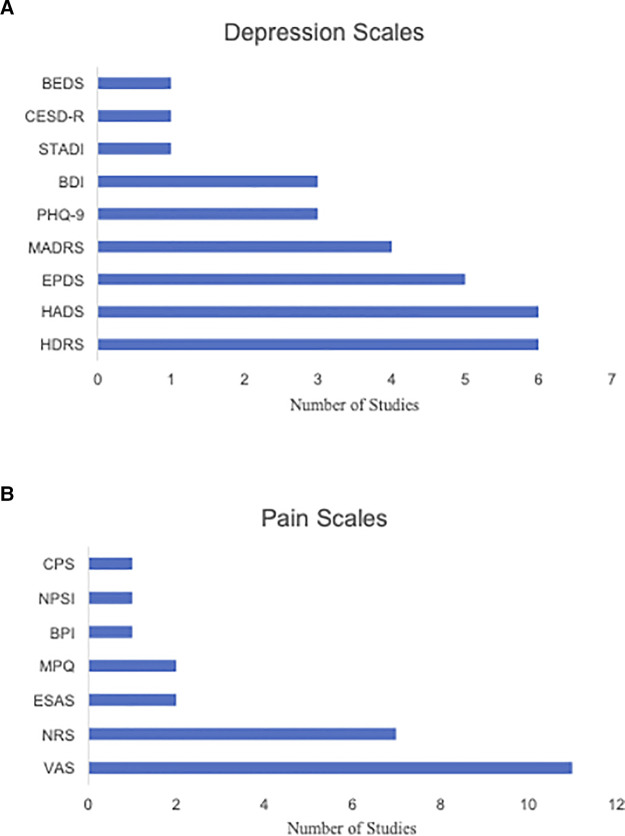
(**A**) Bar chart illustrating the frequency of various depression score measures used across the selected CDCP and CDAP studies. (**B**) Bar chart illustrating the frequency of various pain score measures used across the selected CDCP and CDAP studies. BEDS, Brief Edinburgh Depression Scale; BDI, Beck Depression Inventory; BPI, Brief Pain Inventory; CESD-R, Center for Epidemiological Studies Depression-Revised; CPS, Comparative Pain Scale; ESAS, Edmonton Symptom Assessment System; EPDS, Edinburgh Postnatal Depression Scale; HADS, Hospital Anxiety and Depression Scale; HDRS, Hamilton Depression Rating Scale; MADRS, Montgomery-Asberg Depression Rating Scale; MPQ, McGill Pain Questionnaire; NPSI, Neuropathic Pain Symptom Inventory; NRS, Numerical Rating Scale; PHQ-9, Patient Hospital Questionnaire; STADI, State-Trait Anxiety Depression Inventory; VAS, Visual Analog Scale.

Depression scores in studies assessing CDAP were measured with the following: EPDS, BDI, Brief Edinburgh Depression Scale, Patient Health Questionnaire-9 (PHQ-9), HDRS, and MADRS. Pain was assessed with the following: VAS, NRS, and MPQ. More information about the frequencies of the measures across the published studies can be found in Figures 3A, B.

### 3.6. Findings

#### 3.6.1. Published clinical trials

The findings of the 14 clinical trials (one open-label (51) and 13 randomized controlled trials [RCTs] (39–50, 52)) were heterogeneous in magnitude and duration. Of note, eight of the 14 (57%) clinical trials followed patients for a period of 2 weeks or shorter. Of the 14 clinical trials presented, seven (50%) studied CDCP and seven (50%) studied CDAP.

##### 3.6.1.1. CDCP

Seven studies examined levels of chronic pain symptoms of various aetiologies, including complex regional pain syndrome, cancer-related pain, and refractory chronic pain (39, 40, 42, 44–46, 51). More details on the studies' characteristics and results are presented in Table 1A. Of the seven CDCP studies, three (43%) found a significant decrease in both pain and depression scores (42, 45, 51), with demonstration that high-dose esketamine was more efficacious in reducing pain and depression compared to racemic and low-dose esketamine administration (45). One of these studies, however, demonstrated only a short-term improvement in pain and depression post-hysterectomy in patients with cervical carcinoma (45). Two studies (29%) found a significant decrease in depression scores, with no significant improvement in pain scores (40, 46). One study (14%) found significant improvement in pain but not depression (44). Finally, one study (14%) found no significant improvement in pain or depression scores following ketamine administration (39). This was in the setting of cancer-related chronic pain and depression.

##### 3.6.1.2. CDAP

Seven studies examined pain levels in patients with acute pain symptoms and undergoing surgical procedures (41, 43, 47–50, 52). More details on the studies' characteristics and results are shown in Table 1B. Of the seven CDAP studies, three (43%) found short-term improvement in both depression scores and acute post-operative pain (41, 50, 52). In addition, one study (14%) found a significant decrease in depression symptoms in the short-term period following postpartum caesarean section, however, effects did not sustain at longer time intervals (49). The ketamine group also had significant reductions in pain compared to placebo. A total of three studies found a significant improvement in one of the parameters of either pain scores or mood. Specifically, two studies (29%) found that ketamine only reduced acute pain in the post-operative period, with no effects on mood (47, 48). In contrast, one study (14%) found that ketamine reduced depressive symptoms but had no effect on acute post-operative pain in the setting of postpartum depression following caesarean section (43).

#### 3.6.2. Observational studies

##### 3.6.2.1. CDCP

Corriger and colleagues (54) assessed patients attending pain clinics for refractory chronic pain. It was found that NRS scores decreased from baseline at each of the follow-up checkpoints consisting of one week, six months, and 12 months post-treatment. Similar decreases were found in anxiety and depression subscale scores of the HADS. However, various ketamine dosing regimens were used across the different clinics, and patients were on concomitant treatments for pain. In contrast, Falk and colleagues (53) found that patients in palliative care who received a single low dose (0.25 mg/kg) infusion of esketamine had no significant reductions in pain and depression symptoms when compared to a placebo group. However, the authors' post-hoc power calculations revealed that a sample size of *N* = 20 was needed to reliably determine ketamine efficacy for depression symptoms, suggesting that the power of the study was insufficient.

##### 3.6.2.2. CDAP

Wang and colleagues (55) assessed pain and depression in pregnant women presenting for caesarean section. It was reported that esketamine reduced NRS scores at 2, 4, 8, 24, and 48 h postpartum. In contrast, depression scores were found to be decreased in the esketamine group within 3 months postpartum. Doses administered within the study ranged from 0.2 to 0.5 mg/kg. It was found that NRS scores were lower in the high-dose group (>0.3 mg/kg) than the low-dose group (≤0.3 mg/kg) at 24 and 48 h postpartum. There were no significant differences in depression across the high- and low-dose groups.

#### 3.6.3. Case studies

##### 3.6.3.1. CDCP

Eleven case studies had patients that presented with symptoms of CDCP arising from various conditions, such as chronic regional pain syndrome or cancer-related pain [one (61) of which included two patients]. Ten of the 11 (91%) case studies looking at CDCP demonstrated rapid treatment response and notable reductions in pain and depression symptoms of highly variable duration (56–58, 60, 61, 63–67). In particular, one of these studies reported remarkable reduction in suicide ideation and improvements in affect (61). One additional study described initial improvements in pain levels but later discontinued the treatment due to the onset of manic-like symptoms (59). The remaining case studies indicated, however, that treatment was well-tolerated, with the majority of mild symptoms resolving within minutes or hours after the onset. Additional details on the case studies are included in Table 2.

##### 3.6.3.2. CDAP

The case report carried out by Nichols and colleagues (62) was the only case report which evaluated depression and pain in an acute setting. The patient was treated for severe buttock pain and opioid-induced depressive disorder with intravenous ketamine over 4 days. While there were initial improvements in pain and mood reported on day 4, the patient started to exhibit manic symptoms at which point treatment was discontinued.

#### 3.6.4. Registered ongoing and planned clinical trials

Of the five registered clinical trials, three (60%) (68, 69, 72) were active and recruiting participants, one (20%) (70) was active but not yet recruiting, and one (20%) (71) had a completed status but no published results available. Registered clinical trials that were completed and had their results published were reviewed as “published studies”.

##### 3.6.4.1. CDCP

Two (40%) (70, 71) clinical trials looked at CDCP symptoms, with estimated sample sizes of *N* = 4 and *N* = 80. The origins of the chronic pain were associated with cancer or chronic visceral pain. The ketamine treatments ranged from a dose of 0.125–0.5 mg/kg esketamine given intravenously to an oral 1.0 mg/kg administration given once a day for 12 weeks. Pain outcomes were assessed with the VAS while depression scores were assessed with the HADS, HDRS, and Quick Inventory of Depressive Symptomatology-Self Rated 16-item. More detailed information can be found in Supplementary Table 2.

##### 3.6.4.2. CDAP

Of the five registered trials, three (60%) looked at CDAP (68, 69, 72). In particular, studies looked at acute pain in the setting of surgery-related pain and assessed the presence of MDD symptoms, perioperative, or post-operative depression symptoms. Overall, the planned sample sizes ranged between *N* = 45 and *N* = 564, while treatment doses ranged from 0.2 to 0.5 mg/kg administered intravenously. Pain outcomes were assessed with a rating scale such as the NRS while depression scores were assessed with the MADRS and HADS. More detailed information can be found in the Supplementary Materials.

### 3.7. Adverse effects

**Figure 4:**
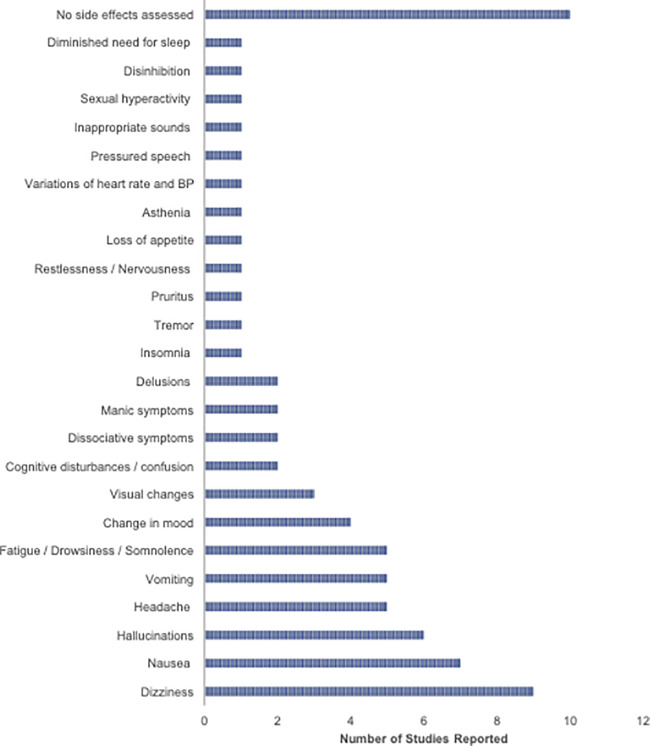
Bar graph illustrates the frequency of adverse effects reported across selected studies looking at CDCP and CDAP. BP, Blood pressure.

There were a variety of acute adverse effects related to ketamine administration that were reported across the included published studies. Common adverse effects included nausea, vomiting, dizziness, and headache. Six out of 29 published studies (43, 47–49, 59, 65) (21%) reported the presence of hallucinations in patients following ketamine administration. The presence of hallucinations was present in both studies looking at CDCP and CDAP. Two of the 29 studies (56, 67) (7%) reported dissociative symptoms. Dissociative symptoms were not reported for any of the studies looking at CDAP. Two case studies assessing CDCP and CDAP reported the discontinuation of ketamine administration after days 3 (59) and 4 (62) of treatment due to the onset of manic symptoms. The adverse effects reported in the participants included inappropriate sounds, sexual hyperactivity, delusions, disinhibition, diminished need for sleep, hallucinations, pressured speech, psychotic thoughts, and euphoric mood. Ten out of 29 studies (34%) did not assess side effects. The distribution of adverse effects across the selected studies is visualized in Figure 4.

## 4. Discussion

### 4.1. Overview of findings

This paper systematically reviewed the literature examining the effects of ketamine administration on CDCP and CDAP symptomatology. Pain and depression symptoms commonly co-occur; for example, patients with depression are more likely to experience back pain while patients with back pain are more likely to experience depression (7, 8). Therefore, there is a great need to address both pain and depression concurrently. Overlapping neurophysiological pathways have been implicated in pain and depression (21), making ketamine, an NMDA-antagonist targeting those pathways, a new promising agent in treating patients with CDCP or CDAP.

Examination of the published literature identified a mixture of studies looking at ketamine efficacy for CDCP as well as CDAP. Therefore, the current review opted to include studies that investigated the effects of ketamine on depression and pain of various aetiologies and types (i.e., acute and chronic). Across all studies investigating CDCP and CDAP, treatment protocols and findings were variable. However, reported adverse effects of ketamine administration were similar across studies looking at both CDCP and CDAP. There were only five registered (ongoing and planned) clinical trials to date: three assessing CDAP and two assessing CDCP.

Forty-three percent of published clinical trials investigating CDCP found significant decreases in pain and depression scores, while the remaining reported conflicting results (i.e., reduction in only depression [29%], pain [14%], or no reduction in either parameter [14%]). One observational study found decreases in chronic pain and depression scores (54), while another found no significant reductions in chronic pain and depression (53). Finally, 91% of the reviewed case studies reported reductions in chronic pain and depression symptoms but the treatment response duration was variable.

Among the published clinical trials investigating CDAP, the results also varied: trials concluded that reductions were found in either acute pain only (29%), depression symptoms only (14%), or both (57%). One observational study reported reductions in depression and pain (55). Similarly, one case study reported initial improvements in both parameters but, following the presence of manic symptoms, treatment was discontinued (62).

Due to heterogeneity in pain conditions, outcome measures, and treatment protocols across the included published studies, a meta-analysis was not considered feasible. This is based on guidelines suggesting that meta-analyses should only be conducted when studies are homogenous in terms of participants, design, and outcomes (73).

### 4.2. Ketamine for CDCP

#### 4.2.1. Published clinical trials

Across published trials, ketamine was found to be efficacious at reducing pain and depression scores in CDCP. The open-label study (51) and two RCTs (42, 45) found large positive effects of intravenous ketamine treatment on the alleviation of depression and pain symptoms in CDCP patients. Interestingly, Wang et al. (45) concluded that reductions in symptoms were significantly greater in the high-dose esketamine group than in the racemic and low-dose esketamine group. Further, short-term (i.e., one and three days post-hysterectomy in cervical carcinoma patients) CDCP symptom improvement differed across single 0.25 mg/kg (pain improvement only) and 0.5 mg/kg (pain and depression improvement) intravenous doses of esketamine (45). Previous research has shown that administration of multiple infusions or high doses resulted in greater improvements and longer-lasting effects in treatment-resistant unipolar and bipolar depression (74), as well as in chronic pain (16). For example, 0.5 mg/kg of ketamine administered intravenously has been found to produce effects lasting up to two weeks in patients with depression (75). Though these effects are still relatively short-term, differences in the type of ketamine administered may explain inconsistencies in the duration of effects, regardless of the dose or route of administration. When comparing esketamine with racemic ketamine, existing research has found that both enantiomers are associated with psychomimetic side effects (76); however, arketamine was found to produce no psychotic symptoms when administered to 10 participants at a dose of 15 mg intravenously in 20 ml of saline compared to a control group of 10 subjects who received esketamine at the same dose (77, 78). Arketamine, when tested in rodents, was also found to be more potent and have longer lasting antidepressant effects than esketamine (79). The present review also found evidence of selective beneficial effects for mood (40, 46) and pain (44) improvement in chronic pain (i.e., no reductions in the other parameter).

Surprisingly, one RCT found orally administered ketamine to be no more effective than placebo at mitigating symptoms of CDCP, including neuropathic pain in cancer (39). The authors proposed that ketamine may be an effective analgesic in subgroups of patients, such as those with central sensitization, that this trial did not focus on exclusively (39). This is supported by previous research which has suggested that, as an NMDA-antagonist, ketamine works by preventing central sensitization and reduces pain hypersensitivity (80), This also raises an important observation that the origins of pain across the selected studies in this review varied. Aetiologies for chronic pain included cancer-related pain, neuropathic pain, and complex regional pain syndrome. These varieties in pain presentation could be a confounding factor in understanding the differences in treatment response duration. The result of this RCT contrasts previous research which has demonstrated that ketamine has significant promise in treating a wide variety of chronic pain conditions, including neuropathic and non-neuropathic pain (16, 17).

#### 4.2.2. Observational studies and case studies

The two observational CDCP studies that were included in the review make it difficult to establish a consensus in terms of findings. Corriger and colleagues (54) employed various routes of administration and dosing regimens (e.g., a majority of participants received ketamine intravenously with cumulative doses of 100–222 mg or 222–270 mg over three to five days) in refractory chronic pain patients. Despite this, pain and depression scores were found to be decreased up to one year following ketamine treatment. This is in line with findings of the published CDCP clinical trials and provides evidence for long-lasting effects of ketamine. In contrast, a retrospective observational study by Falk and colleagues (53) assessed the efficacy of intravenous esketamine in palliative care patients. Inconsistent with the findings of the published RCTs and the first observational study, there were no reported effects of esketamine in reducing either depression or pain symptoms. However, given the small sample size, including eight CDCP patients, and the retrospective design, further research is needed to determine the accuracy of this study's findings.

Overall, CDCP case studies reported a relatively fast treatment response and remarkable reductions in pain and depression symptoms, with variable durations of ketamine effect (e.g., 2 days, 30 days, 3 months). This follows from various treatment regimens, including intravenous, intramuscular, and orally administered ketamine, suggesting that ketamine was generally effective. However, the bioavailability of oral ketamine is low, limiting its clinical use. Oral ketamine has extensive first-pass metabolism with only 17%–24% of oral racemic ketamine and 8%–11% of oral esketamine reaching systemic circulation (81). In contrast, the bioavailability of intravenous ketamine is expected to be 100% (82, 83).

In 50% of the case studies (including one CDAP report) (57, 59–62, 64), changes were qualitatively reported, with no standardized measures used to determine improvement in pain outcomes. When a quantitative assessment was present, the overall formal regime of assessment delivery, symptom monitoring, and regularity of data collection was lacking, thereby limiting the validity of the given findings. Therefore, future studies require rigorous strategies pertaining to assessment, reporting of symptoms, grouping of pain origins, and the course of disorder to better understand treatment outcomes and manifestations.

#### 4.2.3. Registered ongoing and planned clinical trials

The analysis of registered clinical trials for ketamine treatment of CDCP provides insight into the direction wherein this field of research is moving. Though the search results were limited, the two included registered clinical trials were characterized by variability in terms of treatment protocols and patient populations. This is in line with the already published literature and may lead to obstacles when attempting to synthesize the results of the registered trials in prospective reviews. Like the majority of the published trials on the use of ketamine for CDCP, both registered trials were RCTs. Inconsistent with the published literature, ketamine was equally planned to be administered intravenously and orally. Dosages planned to be used in the registered clinical trials were in line with those of the published literature (i.e., ranging from 0.125 to 1.0 mg/kg). While the published clinical trials and observational studies typically employed sample sizes over 100, the two registered trials opted for fewer participants. Taken together, these results suggest that the methodology of upcoming research on the use of ketamine for treatment of CDCP will, for the most part, be similar to that of existing research.

### 4.3. Ketamine for CDAP

#### 4.3.1. Published randomized trials

A significant finding across the examined RCTs is the fast-acting, yet short-lived, effects of ketamine in attenuating depression symptoms and/or acute pain. Six of the seven RCTs reported rapid clinical reductions in acute pain following ketamine administration, with effects being observed within eight hours (52) to five days (41) post-surgery. However, select findings suggest a rather transient nature of ketamine, as its effects on acute pain were no longer significant after one day (52) to one month (49) post-surgery. Furthermore, ketamine produced fast-acting reductions in depression symptoms, with significant effects observed as early as one day (41, 52) to two weeks (50) post-surgery. Notably, ketamine was mainly found to induce transient effects in reducing depression symptoms, as select studies no longer found significant effects after 3 (52) to 42 days (43) post-surgery. These findings are in line with a recent study that observed rapid and transient effects of ketamine in mice, with ketamine infusions alleviating neuropathic pain and depression symptoms for up to 24 h and three days, respectively (84).

Another key observation amongst these studies is the high variability of duration and onset of ketamine efficacy in reducing both (or either) acute pain and depression symptoms in comparison to one another. For example, one study (49) reported that ketamine administration led to a reduction in depression scores one-week post-surgery, whereas significant reductions in pain were reported two days post-surgery. Moreover, another study (47) found that there were no short-term effects of ketamine in reducing either pain or depression symptoms, but reported a delayed effect of ketamine in reducing pain at six weeks post-surgery. Notably, it is difficult to determine whether the variation in ketamine efficacy onset is due to the study design and data recording process, pertaining to a lack of consistent reporting of symptoms overtime, or due to the biological mechanism of ketamine itself.

Furthermore, the RCTs varied in the samples' characteristics, particularly in regard to the severity of their medical conditions: some samples consisted of pregnant women undergoing a caesarean section, while others involved intensive surgeries or life-threatening conditions (i.e., carcinoma patients). Therefore, it's difficult to deduce whether ketamine was equally effective in reducing depression and pain symptoms in each of these populations who varied in the severity of their condition. For example, the sample in Kudoh et al. (52) consisted of MDD patients undergoing orthopedic surgery who experienced transient reductions in acute pain and depression symptoms (≤1 day). In comparison, Han et al. (50) reported longer lasting effects of ketamine in reducing depression symptoms (two weeks postpartum) while Yao et al. (49) reported longer lasting effects of ketamine in reducing pain (two days postpartum) in samples of pregnant women undergoing a caesarean section.

#### 4.3.2. Observational studies and case studies

In line with most RCTs, a single retrospective observational study by Wang et al. (55) determined that esketamine reduced pain and depression following caesarean section. Pain scores, in particular, were found to be lower in the high-dose ketamine (> 0.3 mg/kg) group at 24 and 48 h. This study provides support for the administration of ketamine in CDAP patients as it demonstrated ketamine's potential to also reduce morphine consumption. This is in line with the previous research demonstrating the short- (85) and long-term (86) effects of ketamine on reductions in morphine use post-surgery. Administration of ketamine may, therefore, overcome the problems associated with morphine use (85), such a risk of abuse and addiction.

In contrast, a single case report by Nichols and colleagues (62) investigated ketamine use in a male patient with post-operative pain and opioid-induced depressive disorder. Similar to the results of the included RCTs, ketamine appeared to have transient effects in reducing pain and depression symptoms. However, the patient began to show manic symptoms four days into the ketamine treatment, which resulted in study termination. Long-term effects of ketamine beyond four days are, therefore, inconclusive from the results of this single case study. Importantly, this case study has clinical implications regarding the safety and tolerability of ketamine, as certain individuals may be more prone to experiencing serious adverse effects under ketamine (i.e., mania, delusions).

#### 4.3.3. Registered ongoing and planned clinical trials

Consistent with the designs of the CDAP published studies, the three registered trials plan to utilize similar pain and depression scales (i.e., NRS and MADRS) to measure the efficacy of ketamine in treating acute pain and depression symptoms in post-operative patients. In addition, the registered trials follow a randomized study protocol, which is consistent with the published RCTs. Moreover, the administration route of ketamine in the registered trials is parallel to that of the published trials, as ketamine will be administered intravenously with varied dosing regimens. Given these similarities, the effects of ketamine on CDAP can be better understood by examining a greater number of RCTs that are similar in study design, and therefore, more likely to produce more interpretable and conclusive results.

### 4.4. Strengths and limitations

A strength of the current systematic review is that it included both published and registered clinical trials to provide an up-to-date assessment of research that has been conducted in the field. Furthermore, this review examined research concerning depression co-occurring with acute and chronic pain to gain an understanding of how ketamine may be used to treat comorbid depression and pain of varying aetiologies and durations.

The current review also has a number of limitations. First and foremost, the designs of the included studies were highly heterogeneous: there was a significant variation in inclusion and exclusion criteria of participants, presence of a control group, treatment protocol, primary and secondary measures, and length and frequency of follow-up, which makes it difficult to rule out possible confounding variables. Moreover, some of the studies in the present review (e.g., the retrospective studies, the open-label trial, and case studies) were of limited reliability, validity, and generalizability due to their lack of randomization and/or limited utilization of objective measures. The absence of randomization poses a risk for systematic bias caused by participants' expectations towards the treatment effects. Another important limitation is the high variability in terms of demographics of patient populations, which makes it difficult to hone in on which populations ketamine works most efficiently for. Lastly, another important confounder to consider is various aetiologies of pain included in the various selected studies. Given the diversity in the origins of pain including cancer-related chronic pain, neuropathic pain, or post-operative acute pain, it is difficult to determine whether ketamine is equally efficacious across different pain conditions.

### 4.5. Conclusion and future directions

Across case studies, ketamine was most commonly found to reduce pain and depression symptoms of CDCP. The results of the included published clinical trials and observational studies are mixed, however, as they most commonly suggest that ketamine is effective at reducing both chronic pain and depression or only one of the two comorbidities. Similar to the CDCP literature, research on CDAP suggests that ketamine is effective at reducing both pain and depression, or either parameter individually. Further, the planned methodology of the registered clinical trials for depression and comorbid pain are in line with that of the published research, as they examine pain from various aetiologies with varied dosing regimens across studies. Longer follow-up periods are needed to better understand the long-term effects of ketamine and, in particular, possible changes in the course of CDCP or CDAP, including relapse and remission. Another future avenue for research would be to explore the effects of individual enantiomers on pain and depression. When comparing racemic and esketamine, the reviewed literature found esketamine to be more efficacious in reducing pain; however, one of its limitations was its potential to lead to psychotomimetic side effects. Existing literature suggests that arketamine may be a reasonable alternative with a better side effect profile and longer lasting anti-depressant effects. Future studies should compare esketamine, arketamine, and racemic ketamine at different doses to explore the efficacy of each enantiomer for the treatment of CDCP and CDAP. Studies should also examine whether increasing doses can potentiate effects and produce comparable results between enantiomers, as well as compare the range of side effects in different dose ranges.

## Data Availability

The original contributions presented in the study are included in the article/**Supplementary Material**, further inquiries can be directed to the corresponding author/s.
