# Supplementary material for: Efficacy of ketamine for comorbid depression and acute or chronic pain: A systematic review
Source: Front Pain Res (Lausanne). 2022 Oct 24;3:1022767. doi: 10.3389/fpain.2022.1022767 (PMC9638121; doi:10.3389/fpain.2022.1022767)
Supplement: Supplementary file 1 [file Datasheet1.docx]

***Supplementary Materials***

# Complete search strategy

**Ovid**

1. depression.tw ,kf
2. major depressive disorder.tw ,kf
3. mood disorders*.mp.
4. affective disorders.tw ,kf
5. depress*.mp.
6. pain.tw ,kf
7. neuralgia.tw ,kf
8. postoperative.tw ,kf
9. caesarian section.tw ,kf
10. pain*.mp.
11. 1 OR 2 OR 3 OR 4 OR 5
12. 6 OR 7 OR 8 OR 9 OR 10
13. ketamine.tw ,kf
14. S-ketamine.tw ,kf
15. Esketamine.tw ,kf
16. 13 OR 14 OR 15
17. 11 AND 12 AND 16
18. Remove duplicates from 17
19. Limit 18 to (English language and humans)

**Clinicaltrials.gov**

| **Condition or Disease** | **Intervention** |
| --- | --- |
| (depression OR postpartum depression) AND (pain OR cancer OR surgery) | (ketamine OR S-ketamine OR Esketamine) |

# Supplementary Table 1: GRADE criteria

|  | Selection Bias | Performance Bias | Detection  Bias | Reporting Bias | Selective Bias | End as Scheduled |
| --- | --- | --- | --- | --- | --- | --- |
| Clinical trials and observational studies | | | | | | |
| **Corriger et al** [**(54)**](https://www.zotero.org/google-docs/?GZBk1P)**^C^** |  |  |  |  |  |  |
| **Falk et al** [**(53)**](https://www.zotero.org/google-docs/?TvmIL9)**^C^** |  |  |  |  |  |  |
| **Fallon et al** [**(39)**](https://www.zotero.org/google-docs/?5fsgO1)**^C^** |  |  |  |  |  |  |
| **Han et al** [**(50)**](https://www.zotero.org/google-docs/?rYL3ym)**^A^** |  |  |  |  |  |  |
| **Jafarinia et al** [**(40)**](https://www.zotero.org/google-docs/?HuGd2C)**^C^** |  |  |  |  |  |  |
| **Jiang et al** [**(41)**](https://www.zotero.org/google-docs/?u7WvnX)**^A^** |  |  |  |  |  |  |
| **Kudoh et al** [**(52)**](https://www.zotero.org/google-docs/?SXQLec)**^A^** |  |  |  |  |  |  |
| **Liu et al** [**(42)**](https://www.zotero.org/google-docs/?4zuY5x)**^C^** |  |  |  |  |  |  |
| **Ma et al** [**(43)**](https://www.zotero.org/google-docs/?HFtO0i)**^A^** |  |  |  |  |  |  |
| **Mitchell and Fallon** [**(44)**](https://www.zotero.org/google-docs/?Jim7U1)**^C^** |  |  |  |  |  |  |
| **Sorel et al** [**(51)**](https://www.zotero.org/google-docs/?61AWWI)**^C^** |  |  |  |  |  |  |
| **Wang et al** [**(45)**](https://www.zotero.org/google-docs/?lhQhg8)**^C^** |  |  |  |  |  |  |
| **Wang et al** [**(48)**](https://www.zotero.org/google-docs/?KvL2BZ)**^A^** |  |  |  |  |  |  |
| **Wang et al** [**(55)**](https://www.zotero.org/google-docs/?HOScOR)**^A^** |  |  |  |  |  |  |
| **Xu et al** [**(46)**](https://www.zotero.org/google-docs/?eHVUCs)**^C^** |  |  |  |  |  |  |
| **Xu et al** [**(47)**](https://www.zotero.org/google-docs/?rOM4EL)**^A^** |  |  |  |  |  |  |
| **Yao et al** [**(49)**](https://www.zotero.org/google-docs/?nz29Nx)**^A^** |  |  |  |  |  |  |
| Case studies | | | | | | |
| **Barbosa et al (2020)** [**(56)**](https://www.zotero.org/google-docs/?RqMvcD)**^C^** |  |  |  |  |  |  |
| **Bigman et al (2017)** [**(57)**](https://www.zotero.org/google-docs/?fzSNkK)**^C^** |  |  |  |  |  |  |
| **Hanna et al (2017)** [**(58)**](https://www.zotero.org/google-docs/?MWNgus)**^C^** |  |  |  |  |  |  |
| **Mandyam and Ahuja (2017)** [**(59)**](https://www.zotero.org/google-docs/?MKyr5g)**^C^** |  |  |  |  |  |  |
| **McNulty and Hahn (2012)** [**(60)**](https://www.zotero.org/google-docs/?eB0ii0)**^C^** |  |  |  |  |  |  |
| **Mischel et al (2018)** [**(61)**](https://www.zotero.org/google-docs/?P6asLN)**^C^** |  |  |  |  |  |  |
| **Nichols et al (2016)** [**(62)**](https://www.zotero.org/google-docs/?ntiUe2)**^A^** |  |  |  |  |  |  |
| **Rodríguez-Mayoral et al (2020)** [**(63)**](https://www.zotero.org/google-docs/?cfXewn)**^C^** |  |  |  |  |  |  |
| **Sexton et al (2018)** [**(64)**](https://www.zotero.org/google-docs/?pqQjtQ)**^C^** |  |  |  |  |  |  |
| **Stefanczyk-Sapieha et al (2008)** [**(65)**](https://www.zotero.org/google-docs/?iUMxx3)**^C^** |  |  |  |  |  |  |
| **Weber et al (2018)** [**(66)**](https://www.zotero.org/google-docs/?rgK9qO)**^C^** |  |  |  |  |  |  |
| **Zanicotti et al (2012)** [**(67)**](https://www.zotero.org/google-docs/?dQADDS)**^C^** |  |  |  |  |  |  |

**Abbreviations:** A= Acute, categorization of studies assessing acute pain; C= Chronic, categorization of studies assessing chronic pain

**Legend:** Green square= low risk, GRADE criteria met; red square= high risk, GRADE criteria not met; grey square= not applicable (case study) or not reported

# 3 Supplementary Table 2: Selected characteristics of studies registered on Clinicaltrials.gov

| PI (Clinical Trial Identifier) | Sample Size | Diagnosis | Study Design | Dose/Route | Outcome Parameters | Start and Reg Date | Estimated/Actual Completion Date (Status) |
| --- | --- | --- | --- | --- | --- | --- | --- |
| **Han, R.**  **(NCT04425473) (72)^A^** | 564 | Perioperative depression | Double blind RCT | 0.2 mg/kg IV esketamine when suturing incision | 1. Primary: MADRS (depression score)  2. Secondary: rate of severe pain after surgery | Start Date: February 2021  Reg Date: June 2020 | December 31, 2023 (Recruiting) |
| **Heifets, B.**  **(NCT03861988) (69)^A^** | 45 | MDD undergoing total joint replacement surgery | Open label and double-blind RCT | 0.5 mg/kg IV ketamine during surgery | 1. Primary: HADS (depression score)  2. Primary: MADRS (depression score)  3. Secondary: rate of pain after surgery | Start date: August 2019  Reg Date: March 2019 | January 2022  (Recruiting) |
| **Irwin, S. (NCT02836288) (71)^C^** | 4 | Cancer and depression | Double blind RCT | 1.0 mg/kg oral ketamine | 1. Secondary: QIDS-SR-16 (depression score)  2. Secondary: VAS (pain score)  3. Secondary: HADS (depression score) | Start Date: December 2016  Reg Date: July 2016 | May 29, 2018  (Completed) |
| **Tianmei, S.**  **(NCT04847245) (70)^C^** | 80 | Chronic visceral pain comorbid MDD | Single-blind RCT | IV esketamine with doses:  1. 0.125 mg/kg  2. 0.25 mg/kg  3. 0.5 mg/kg | 1. Primary: VAS (pain levels)  2. Secondary: HDRS (depression score) | Reg Date: April 2021 | March 1, 2023  (Not yet recruiting) |
| **Wu, A.**  **(NCT05155969) (68)^A^** | 80 | Cardiac surgery post-operative depression | Double blind RCT | 0.3 mg/kg IV esketamine before anesthesia induction | 1. Primary: MADRS (depression score)  2. Secondary: NRS (pain score) | Start Date: December 2021  Reg Date: December 2021 | December 15, 2022 (Recruiting) |

**Abbreviations:** A= Acute, categorization of studies assessing acute pain; C= Chronic, categorization of studies assessing chronic pain; HADS= Hospital Anxiety and Depression Scale; HDRS= Hamilton Depression Rating Scale; IV= Intravenous; MADRS= Montgomery-Åsberg Depression Rating Scales; MDD= Major Depressive Disorder; NRS= Numerical Rating Scale; PI= Principal Investigator; QIDS-SR-16= Quick Inventory of Depressive Symptomatology-Self Rated 16-item; RCT= Randomized Controlled Trial; Reg= Registration; VAS= Visual Analog Scale

# 4 Supplementary Table 3: PRISMA abstract checklist

| **Topic** | **No.** | **Item** | **Reported?** |
| --- | --- | --- | --- |
| **TITLE** |  |  |  |
| Title | 1 | Identify the report as a systematic review. | Yes |
| **BACKGROUND** |  |  |  |
| Objectives | 2 | Provide an explicit statement of the main objective(s) or question(s) the review addresses. | Yes |
| **METHODS** |  |  |  |
| Eligibility criteria | 3 | Specify the inclusion and exclusion criteria for the review. | Yes |
| Information sources | 4 | Specify the information sources (e.g. databases, registers) used to identify studies and the date when each was last searched. | Yes |
| Risk of bias | 5 | Specify the methods used to assess risk of bias in the included studies. | Yes |
| Synthesis of results | 6 | Specify the methods used to present and synthesize results. | N/A |
| **RESULTS** |  |  |  |
| Included studies | 7 | Give the total number of included studies and participants and summarise relevant characteristics of studies. | Yes |
| Synthesis of results | 8 | Present results for main outcomes, preferably indicating the number of included studies and participants for each. If meta-analysis was done, report the summary estimate and confidence/credible interval. If comparing groups, indicate the direction of the effect (i.e. which group is favoured). | Yes |
| **DISCUSSION** |  |  |  |
| **Limitations of evidence** | 9 | Provide a brief summary of the limitations of the evidence included in the review (e.g. study risk of bias, inconsistency and imprecision). | Yes |
| **Interpretation** | 10 | Provide a general interpretation of the results and important implications. | Yes |
| **OTHER** |  |  |  |
| **Funding** | 11 | Specify the primary source of funding for the review. | N/A |
| **Registration** | 12 | Provide the register name and registration number. | N/A |

*From:* Page MJ, McKenzie JE, Bossuyt PM, Boutron I, Hoffmann TC, Mulrow CD, et al. The PRISMA 2020 statement: an updated guideline for reporting systematic reviews. MetaArXiv. 2020, September 14. DOI: 10.31222/osf.io/v7gm2. For more information, visit: [www.prisma-statement.org](http://www.prisma-statement.org)

#

# 5 Supplementary Table 4: PRISMA checklist

| **Section and**  **Topic** | **Item** | **Checklist item** | **Location where reported** |
| --- | --- | --- | --- |
| **TITLE** | | |  |
| Title | 1 | Identify the report as a systematic review. | Page 1, title |
| **ABSTRACT** | | |  |
| Abstract | 2 | See the PRISMA Abstracts checklist above. | Page 1-2, lines 38-62 |
| **INTRODUCTION** | | |  |
| Rationale | 3 | Describe the rationale for the review in the context of existing knowledge. | Pages 3-4, lines 79-151 |
| Objectives | 4 | Provide an explicit statement of the objective(s) or question(s) the review addresses. | Page 4, lines 140-142 |
| **METHODS** | | |  |
| Eligibility criteria | 5 | Specify the inclusion and exclusion criteria for the review and how studies were grouped for the syntheses. | Page 5, lines 178-196 |
| Information  sources | 6 | Specify all databases, registers, websites, organisations, reference lists and other sources searched or consulted to identify studies. Specify the date when each source was last searched or consulted. | Pages 4-5, lines 158-176 |
| Search strategy | 7 | Present the full search strategies for all databases, registers and websites, including any filters and limits used. | Supplementary Materials |
| Selection process | 8 | Specify the methods used to decide whether a study met the inclusion criteria of the review, including how many reviewers screened each record and each report retrieved, whether they worked independently, and if applicable, details of automation tools used in the process. | Page 5, lines 167-170 |
| Data collection  process | 9 | Specify the methods used to collect data from reports, including how many reviewers collected data from each report, whether they worked independently, any processes for obtaining or confirming data from study investigators, and if applicable, details of automation tools used in the process. | Page 5, lines 198-199 |
| Data items | 10a | List and define all outcomes for which data were sought. Specify whether all results that were compatible with each outcome domain in each study were sought (e.g. for all measures, time points, analyses), and if not, the methods used to decide which results to collect. | Page 5-6, lines 198-217 |
|  | 10b | List and define all other variables for which data were sought (e.g. participant and intervention characteristics, funding sources). Describe any assumptions made about any missing or unclear information. | Page 5-6, lines 198-217 |
| Study risk of bias assessment | 11 | Specify the methods used to assess risk of bias in the included studies, including details of the tool(s) used, how many reviewers assessed each study and whether they worked independently, and if applicable, details of automation tools used in the process. | Pages 6, lines 219-224 |
| Effect measures | 12 | Specify for each outcome the effect measure(s) (e.g. risk ratio, mean difference) used in the synthesis or presentation of results. | N/A |
| Synthesis  methods | 13a | Describe the processes used to decide which studies were eligible for each synthesis (e.g. tabulating the study intervention characteristics and comparing against the planned groups for each synthesis (item #5)). | Page 5, lines 178-196 |
|  | 13b | Describe any methods required to prepare the data for presentation or synthesis, such as handling of missing summary statistics, or data conversions. | N/A |
|  | 13c | Describe any methods used to tabulate or visually display results of individual studies and syntheses. | Tables 1 & 2 |
|  | 13d | Describe any methods used to synthesize results and provide a rationale for the choice(s). If meta-analysis was performed, describe the model(s), method(s) to identify the presence and extent of statistical heterogeneity, and software package(s) used. | Tables 1 & 2 |
|  | 13e | Describe any methods used to explore possible causes of heterogeneity among study results (e.g. subgroup analysis, meta-regression). | Page 12, lines 485-488 |
|  | 13f | Describe any sensitivity analyses conducted to assess robustness of the synthesized results. | N/A |
| Reporting bias  assessment | 14 | Describe any methods used to assess risk of bias due to missing results in a synthesis (arising from reporting biases). | N/A |
| Certainty  assessment | 15 | Describe any methods used to assess certainty (or confidence) in the body of evidence for an outcome. | N/A |

| **RESULTS** | | |  |
| --- | --- | --- | --- |
| Study selection | 16a | Describe the results of the search and selection process, from the number of records identified in the search to the number of studies included in the review, ideally using a flow diagram. | Page 6, line 238-248 & Figure 1 |
|  | 16b | Cite studies that might appear to meet the inclusion criteria, but which were excluded, and explain why they were excluded. | N/A |
| Study  characteristics | 17 | Cite each included study and present its characteristics. | Tables 1 & 2 |
| Risk of bias in  studies | 18 | Present assessments of risk of bias for each included study. | Supplementary Materials |
| Results of  individual studies | 19 | For all outcomes, present, for each study: (a) summary statistics for each group (where appropriate) and (b) an effect estimate and its precision (e.g. confidence/credible interval), ideally using structured tables or plots. | Tables 1 & 2 |
| Results of  syntheses | 20a | For each synthesis, briefly summarise the characteristics and risk of bias among contributing studies. | Page 6 line 225-235 & Supplementary Materials |
|  | 20b | Present results of all statistical syntheses conducted. If meta-analysis was done, present for each the summary estimate and its precision (e.g.  confidence/credible interval) and measures of statistical heterogeneity. If comparing groups, describe the direction of the effect. | N/A |
|  | 20c | Present results of all investigations of possible causes of heterogeneity among study results. | Page 6-11, line 251-451 & Supplementary Materials |
|  | 20d | Present results of all sensitivity analyses conducted to assess the robustness of the synthesized results. | N/A |
| Reporting biases | 21 | Present assessments of risk of bias due to missing results (arising from reporting biases) for each synthesis assessed. | N/A |
| Certainty of  evidence | 22 | Present assessments of certainty (or confidence) in the body of evidence for each outcome assessed. | N/A |
| **DISCUSSION** | | |  |
| Discussion | 23a | Provide a general interpretation of the results in the context of other evidence. | Page 12-16, line 491-640 |
|  | 23b | Discuss any limitations of the evidence included in the review. | Table 1 & Page 16, line 647-661 |
|  | 23c | Discuss any limitations of the review processes used. | Page 16, line 647-661 |
|  | 23d | Discuss implications of the results for practice, policy, and future research. | Page 16-17, line 663-683 |
| **OTHER INFORMATION** | | |  |
| Registration and  protocol | 24a | Provide registration information for the review, including register name and registration number, or state that the review was not registered. | N/A |
|  | 24b | Indicate where the review protocol can be accessed, or state that a protocol was not prepared. | N/A |
|  | 24c | Describe and explain any amendments to information provided at registration or in the protocol. | N/A |
| Support | 25 | Describe sources of financial or non-financial support for the review, and the role of the funders or sponsors in the review. | Page 17, line 705 |
| Competing  interests | 26 | Declare any competing interests of review authors. | Page 17, line 685-693 |
| Availability of  data, code and  other materials | 27 | Report which of the following are publicly available and where they can be found: template data collection forms; data extracted from included studies; data used for all analyses; analytic code; any other materials used in the review. | Page 24, line 992-993 |

*From:* Page MJ, McKenzie JE, Bossuyt PM, Boutron I, Hoffmann TC, Mulrow CD, et al. The PRISMA 2020 statement: an updated guideline for reporting systematic reviews. BMJ 2021;372:n71. doi:  10.1136/bmj.n71 For more information, visit: [www.prisma-statement.org](http://www.prisma-statement.org)
